# Detection of potential functional variants based on systems-biology: the case of feed efficiency in beef cattle

**DOI:** 10.1186/s12864-022-08958-y

**Published:** 2022-11-25

**Authors:** Gabriela Ribeiro, Fernando Baldi, Aline S. M. Cesar, Pâmela A. Alexandre, Elisa Peripolli, José B. S. Ferraz, Heidge Fukumasu

**Affiliations:** 1 Department of Veterinary Medicine, Faculty of Animal Science and Food Engineering, University of Sao Paulo, Pirassununga, Sao Paulo, 13635-900 Brazil; 2 Department of Animal Science, São Paulo State University (UNESP), Jaboticabal, São Paulo, Brazil; 3 Escola Superior de Agricultura “Luiz de Queiroz”, University of Sao Paulo, Piracicaba, São Paulo, Brazil; 4 CSIRO Agriculture & Food, 306 Carmody Rd., St. Lucia, Brisbane, QLD 4067 Australia

**Keywords:** Functional variants, Transcriptomics, GWAS, Cattle, Feed efficiency

## Abstract

**Background:**

Potential functional variants (PFVs) can be defined as genetic variants responsible for a given phenotype. Ultimately, these are the best DNA markers for animal breeding and selection, especially for polygenic and complex phenotypes. Herein, we described the identification of PFVs for complex phenotypes (in this case, Feed Efficiency in beef cattle) using a systems-biology driven approach based on RNA-seq data from physiologically relevant organs.

**Results:**

The systems-biology coupled with deep molecular phenotyping by RNA-seq of liver, muscle, hypothalamus, pituitary, and adrenal glands of animals with high and low feed efficiency (FE) measured by residual feed intake (RFI) identified 2,000,936 uniquely variants. Among them, 9986 variants were significantly associated with FE and only 78 had a high impact on protein expression and were considered as PFVs. A set of 169 significant uniquely variants were expressed in all five organs, however, only 27 variants had a moderate impact and none of them a had high impact on protein expression. These results provide evidence of tissue-specific effects of high-impact PFVs. The PFVs were enriched (FDR < 0.05) for processing and presentation of MHC Class I and II mediated antigens, which are an important part of the adaptive immune response. The experimental validation of these PFVs was demonstrated by the increased prediction accuracy for RFI using the weighted G matrix (ssGBLUP+wG; Acc = 0.10 and b = 0.48) obtained in the ssGWAS in comparison to the unweighted G matrix (ssGBLUP; Acc = 0.29 and b = 1.10).

**Conclusion:**

Here we identified PFVs for FE in beef cattle using a strategy based on systems-biology and deep molecular phenotyping. This approach has great potential to be used in genetic prediction programs, especially for polygenic phenotypes.

**Supplementary Information:**

The online version contains supplementary material available at 10.1186/s12864-022-08958-y.

## Background

Potential functional variants (PFVs) can be defined as genetic variants responsible for a given phenotype. Ultimately, these are the DNA markers needed for animal breeding and selection specially for polygenic and complex phenotypes [1]. One possibility to detect PFVs is the analysis of whole-genome sequencing (WGS), an approach that is still very costly. Another possibility is the whole-exome (WES) sequencing which is not as expensive as the WGS, however, it still lacks information regarding the importance of DNA variants within the cell and/or tissue architecture related to phenotypes [2]. As the phenotype is nothing more than a coordinate set of genes being expressed, one should have in mind that a complex phenotype is made by contributions of several cell types, organs, and their interactions [3]. Therefore, an organ-level systems biology approach should be considered since it will point out specific DNA variants associated with relevant biological processes for specific phenotypes. Hence, it is feasible to consider approaches that integrate the information of functional genes in relevant organs to find PFVs for future animal breeding and selection purposes.

Feed efficiency (FE) in beef cattle is one of the most important traits in livestock [4]. While beef cattle produce high-quality meat from low-quality forage, they are one of the least efficient animals at converting feed into protein [5], being recognized as one of the largest contributors to greenhouse gas emissions [6]. Therefore, more efficient animals are highly needed worldwide since their improved productivity and sustainability can reduce production costs, which can reach up to 75% of the income expenses in feedlot systems [7]. Further such efficient animals can also decrease methane production, one of the greenhouse gases, reducing the impact on the environment [8, 9]. It should be noted that identifying high FE animals is not an easy task as it is a complex phenotype that is controlled by several interconnected mechanisms [10, 11]. Thus, it is necessary to understand the biological basis of FE to define future animal breeding programs [12].

Our research group was the first to describe a pathophysiological mechanism associated with FE in beef cattle: liver inflammation due to altered metabolism and/or bacterial translocation/infection [13], which was partially corroborated by others [14–16]. We also unraveled the metabolic pathways related to FE in Nellore cattle showing an increased bacterial load in low feed efficient animals, which is in part, responsible for the hepatic lesions and inflammation in such animals [17]. Previously, some QTLs for FE in Nellore beef cattle were found through conventional GWAS [18–22], however only attempts to find causal variants were made. Therefore, we propose a system-biology strategy to overcome these limitations based on the identification of genetic variants from RNA-sequencing (RNA-seq) data from physiologically phenotype-related organs, followed by a classification of the PFVs according to their effects on protein expression and function. We also validated the potential functional variants through differential weighting genomic regions harboring PFVs for genomic prediction of RFI in a different non-related population.

## Results

### Detection and characterization of potential functional variants (PFVs) associated with feed efficiency

**Fig. 1:**
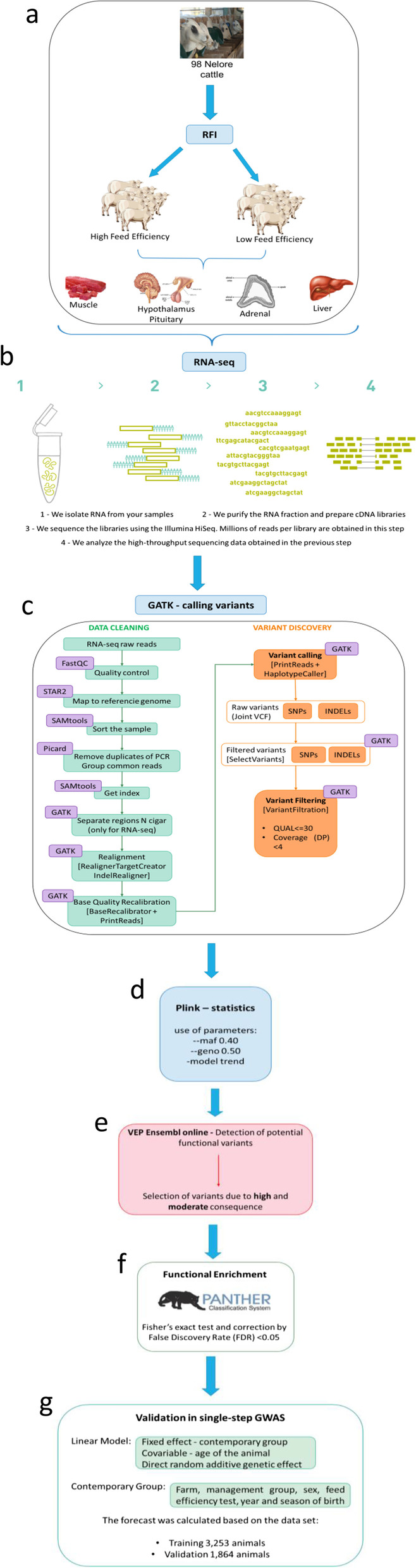
The pipeline for PFV detection. Step-by-step details of the material and methods for obtaining potential causal variants. **a** Selection of animals for feed efficiency and sample preparation; **b** RNA-seq analysis (paired end); **c** Data treatment and call for variants by the GATK tool; **d** Statistical analysis and genetic association using Plink; **e** Identification of the impact and consequence of variants by the Ensembl VEP online; **f** Functional enrichment using Panther (GO); **g** Validation of results using the GWAS of an independent population

**Fig. 2:**
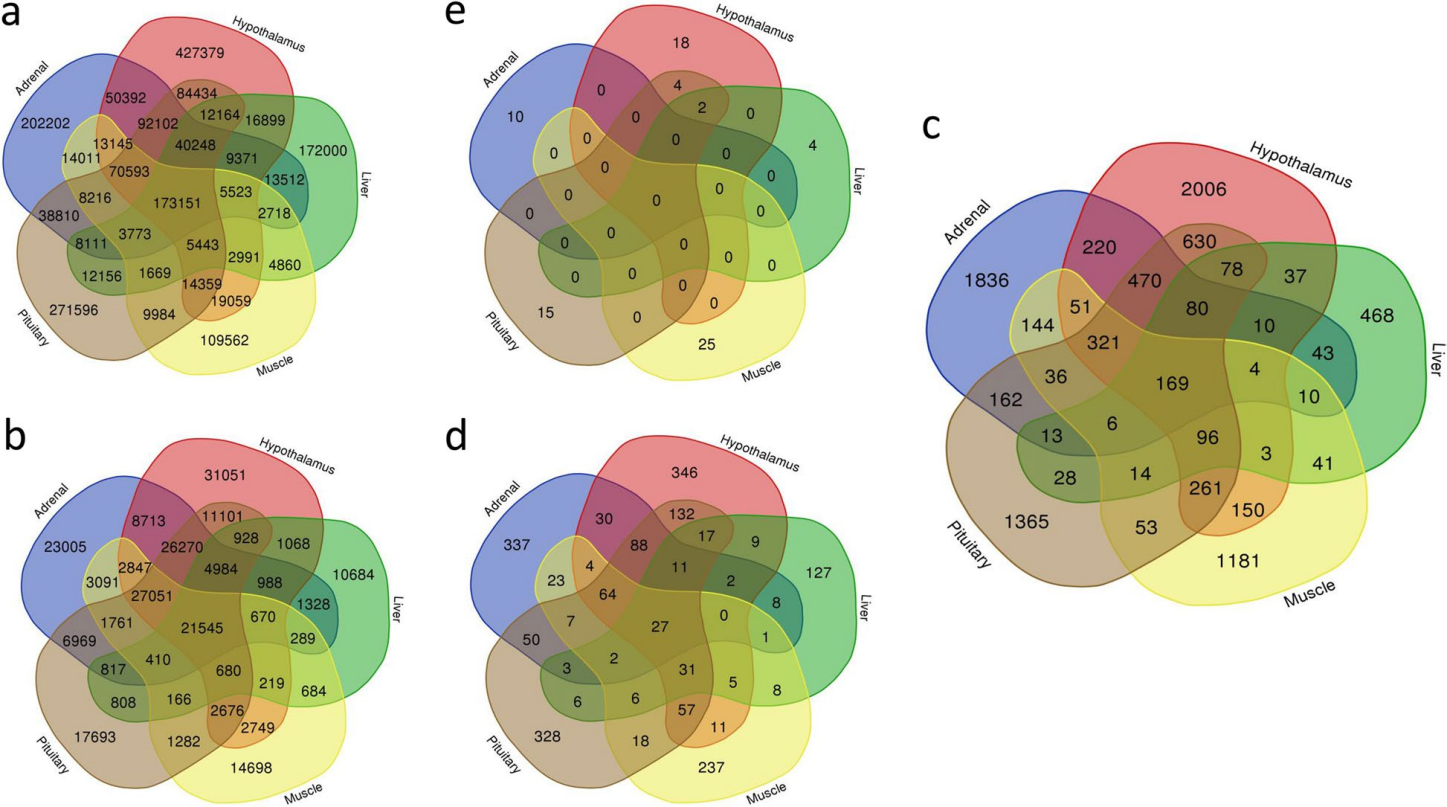
Variants overlay (SNPs and INDELs). **a** Number of SNPs and INDELs variants (insertions and deletions), **b** Variants filtered according to maf < 0.40 and call rate 0.50 and associated with the genotype; **c** Significant variations associated with feed efficiency; **d** Potential functional variants with moderate impact, in other words, a non-disruptive variant that can alter the effectiveness of the protein; **e** Potential functional variants with high impact, causing protein truncation and loss of function

The strategy proposed here (Fig. 1) is to detect PFVs based on systems biology and deep phenotyping by RNA-seq of relevant organs for a given phenotype, in this case, FE in beef cattle. For this experiment, we used samples from nine animals of each group (HFE and LFE), analyzing 18 samples of liver, hypothalamus, and pituitary glands; 17 of muscle and 15 of the adrenal glands, yielding 13,3 million reads per sample, on average (Table 1 and Additional file 1). Initially, variants were called from the five different organs, and the number of unique variants was 2,000,936 due to the overlap of variant calling in such organs (Table 1). After filtering the variants by MAF and call rate, a total of 11,35% (*n = 227,225* unique variants) was used for statistical analysis, in which 4,39% (*n = 9986* variants) were significantly associated with FE. Next, we classified the PFVs according to their impact on protein function, in which 20,0% *(n = 1995* variants) were classified with moderate impact and only 0.78% (*n = 78* variants) with high impact on protein function (Table 1 and Fig. 2). The majority of the PFVs with moderate or high impact are missense SNPs (Table 2 and Fig. 2), however, there are other relevant protein consequences as frameshift INDELs, stop gained INDELs, and inframe insertion INDELs which alter protein sequences and function.

**Table 1:** Number of variants called for feed efficiency in Nellore beef cattle from the five different organs

Organ	Total variant calling	Variants after filtering^b^	Significant variants	Impact on protein function	
				Moderate	High
Liver	484,589	46,268	1110	263	6
Muscle	459,057	80,818	2540	501	25
Hypothalamus	1,037,253	143,540	4586	834	24
Pituitary	846,809	125,141	3782	847	21
Adrenal	745,878	130,738	3575	657	10
Total	3,573,586	526,505	15,593	3102	86
Uniquely variants^a^	2,000,936	227,225	9986	1995	78

The results of the Total variant number, Filter, and Significant variants are in quantities, in other words, the number of variants that can be SNPs or/and INDELs. ^a^ This data reports the number of uniquely variants since there are variants detected in more than one tissue. ^b^ Variants were filtered for MAF and call rate

**Table 2:** Variants distributed according to the consequences on protein sequence

Consequence ^a^													
Organ	Missense	Splice region	Splice donor	Frameshift	Stop gained	Inframe insertion	Inframe deletion	Splice acceptor	Start lost	Stop lost	Intron variant	Start retained	Affected genes
Liver	260	2	3	3	1	3	–	–	–	–	–	–	120
Muscle	494	24	8	14	–	3	4	1	1	1	–	–	211
Hypothalamus	818	11	11	10	3	10	9	–	–	–	–	–	352
Pituitary	823	9	5	12	3	10	17	–	2	1	–	2	343
Adrenal	635	6	2	3	2	9	15	3	–	–	2	–	286
Total	3030	52	29	42	9	35	45	4	3	2	2	2	1312

^a^ The number of consequences of the variants can vary, as a variant can have more than one consequence

Interestingly, we found 169 significant variants expressed in all five organs (Fig. 2c), however, only 27 of them displayed moderate impact and none high impact on protein function. These results provide evidence of tissue-specific effects of high-impact functional variants. In addition, the pituitary glands and the hypothalamus ranked as the first and second organs with the most significant variants in our study. These 27 variants were located close to genes related to the process of apoptosis, oxidation, transcription factors, interferons, DNA repair, rRNA, and tRNA processing (Table 3).

**Table 3:** Variants with moderate impact on protein function described in all five tissues

Symbol	ID Genes	Variant Name	Protein Position	SNP	Protein Alteration
COL4A1	ENSBTAG00000012849	ENSBTAT00000076567	932	C	T/A
COL4A1	ENSBTAG00000012849	ENSBTAT00000035335	1013	C	T/A
ECHS1	ENSBTAG00000017710	ENSBTAT00000079039	171	C	F/L
ECHS1	ENSBTAG00000017710	ENSBTAT00000044947	106	C	F/L
ECHS1	ENSBTAG00000017710	ENSBTAT00000082162	155	C	F/L
ERCC5	ENSBTAG00000014043	ENSBTAT00000046365	1132	C	V/A
ERCC5	ENSBTAG00000014043	ENSBTAT00000046365	1132	G	P/A
ETS2	ENSBTAG00000009214	ENSBTAT00000012144	231	C	I/L
ETS2	ENSBTAG00000009214	ENSBTAT00000074510	231	C	I/L
FAM207A	ENSBTAG00000017010	ENSBTAT00000022620	150	C	W/R
FAM207A	ENSBTAG00000017010	ENSBTAT00000022620	155	G	P/R
IRF3	ENSBTAG00000006633	ENSBTAT00000031869	162	C	N/K
MTCH2	ENSBTAG00000018742	ENSBTAT00000024956	82	C	I/V
MTCH2	ENSBTAG00000018742	ENSBTAT00000084129	82	C	I/V
NBN	ENSBTAG00000013225	ENSBTAT00000017598	709	G	M/V
NBN	ENSBTAG00000013225	ENSBTAT00000017598	693	T	P/S
NBN	ENSBTAG00000013225	ENSBTAT00000017598	686	C	V/L
NUBP1	ENSBTAG00000009560	ENSBTAT00000012576	224	A	V/I
OSGIN2	ENSBTAG00000013678	ENSBTAT00000018177	245	C	N/D
RPL7L1	ENSBTAG00000018478	ENSBTAT00000005214	121	C	G/R
RPL7L1	ENSBTAG00000018478	ENSBTAT00000073451	108	C	G/R
RPL7L1	ENSBTAG00000018478	ENSBTAT00000005214	48	A	R/K
RPL7L1	ENSBTAG00000018478	ENSBTAT00000073451	35	A	R/K
RPP30	ENSBTAG00000002973	ENSBTAT00000003871	252	AGC	E/EA
RPP30	ENSBTAG00000002973	ENSBTAT00000084821	250	AGC	E/EA
SCAND1	ENSBTAG00000005573	ENSBTAT00000007322	75	T	V/I
UTP3	ENSBTAG00000009310	ENSBTAT00000012263	305	A	R/K

Twenty-seven functional variants with moderate impact associated with feed efficiency in Nellore cattle presented in five tissues studied (liver, adrenal, hypothalamus, pituitary glands, and muscle). These variants are non-disruptive that can alter the effectiveness of proteins

### Functional analysis of the PFVs

Functional enrichment was performed in two scenarios: (i) first with all the genes carrying the PFVs grouped, disregarding which tissues they came from, and second (ii) by considering the PFVs separately for each tissue. In the first scenario, all enriched terms were related to the processing and presentation of MHC Class I and MHC class II mediated antigens (major histocompatibility complex), which are an essential mechanism of the adaptive immune response. In addition to the innate immunity system enriched terms, vesicle-mediated transport, cell signaling, ubiquitination, double-strand break repair, and nucleotide excision were also reported.

The tissue-specific enrichment analysis mainly described terms related to the processing and presentation of MHC Class I and MHC class II antigens, innate immune system, vesicle-mediated transport, cell signaling, ubiquitination, and DNA double-strand break repair. It should be highlighted that the RNA polymerase III transcription pathway was also indicated in the adrenal glands, muscle, hypothalamus, and liver. The simple DNA strand repair pathway was enriched in the muscle and the nucleotide excision pathway was over-represented in the adrenal glands, hypothalamus, and liver tissues (Additional file 2). The list of these 20 genes, their potential functional variants and impact on the protein sequence, and the frequency in each group can be found in Additional file 2.

### Validation of the findings using genomic prediction

A total of 144, 252, 413, 416, and 340 PFVs identified in the liver, muscle, hypothalamus, pituitary, and adrenal glands were adjacent to 223, 422, 694, 697, and 554 SNP markers presented in the BovineHD (Illumina), respectively. To carry out the validation and genomic predictions for the validation set, SNP markers adjacent to the PFVs were differentially weighted based on the results obtained with the ssGWAS using the training dataset. The genomic prediction ability for the RFI in the validation dataset when the PFVs information is not included in the analyses is shown in Table 4. The prediction accuracy for the RFI using the weighted G matrix (ssGBLUP+wG) obtained in the ssGWAS of the training dataset was higher than the unweighted G matrix (ssGBLUP). However, the prediction accuracy enhancement was higher when RFI records were added (ssGBLUP+rec) in the validation subset compared to applying a weighted G matrix (Table 4). As expected, the highest prediction accuracy in the validation dataset was obtained when all available information was considered together with the weighted G matrix (ssGBLUP+wG + rec), however, more inflated predictions for RFI were obtained. By also applying the ssGBLUP method [23], (0.45) and [24] (0.22) reported higher prediction ability for RFI in Nellore cattle. The less inflated predictions for RFI were obtained with the model that includes unweighted genomic information and records of the validation dataset, however, in this scenario, phenotypic information is necessary. It is important to highlight that the more realistic scenario is to use the genomic information to predict the GEBV of young animals without RFI records at early ages to maximize the genetic progress for RFI and take advantage of the genomic selection. It should be noted that the availability of phenotypic records for RFI is not common in beef cattle breeding programs since it is expensive to measure.

**Table 4:** Prediction ability (Acc) and regression coefficient (b) for residual feed intake in the validation set

Validation without PFVs^a^	Acc	b (SE)
ssGBLUP	0.10	0.48 (0.02)
ssGBLUP+wG	0.15	0.85 (0.03)
ssGBLUP+records	0.23	1.00 (0.03)
ssGBLUP+wG + records	0.29	1.10 (0.02)

^a^*ssGBLUP* ssGBLUP using unweighted G matrix, *ssGBLUP + wG* ssGBLUP using weighted G matrix, *ssGBLUP + records* ssGBLUP using unweighted G matrix and records, *ssGBLUP + wG + records* ssGBLUP using weighted G matrix and records

The prediction ability for each tissue improved in comparison to the ssGBLUP+wG by adding the information of the PFVs with differentially weighted SNPs (Table 5). The prediction ability for RFI using 1-fold, 2-fold, and 3-fold were almost the same for the different tissues, however, the highest prediction accuracy was obtained in the 3-fold scenario, in which the prediction accuracy increased from 31.03 to 40% compared to the weighted G matrix without considering the PFVs. Despite the high prediction accuracy for RFI when SNP markers adjacent to the PFVs were differentially weighted, more inflated predictions were obtained for RFI as the weighted for the PFVs increased. However, it is important to highlight that the increase of the prediction inflation was lower in the liver and muscle tissue compared to the adrenal glands, pituitary, or hypothalamus.

**Table 5:** Prediction ability (Acc) and regression coefficient (b) for RFI differentially (SNPs and PFV)

Validation for functional mutations	Adrenal		Pituitary		Hypothalamus		Muscle		Liver	
	Acc	b (SE)	Acc	b (SE)	Acc	b (SE)	Acc	b (SE)	Acc	b (SE)
**ssGBLUP + wG + QTN:1-fold**	0.16	0.74 (0.03)	0.16	0.723 (0.03)	0.15	0.73 (0.03)	0.15	0.85 (0.03)	0.15	0.82 (0.03)
**ssGBLUP + wG + QTN:2-fold**	0.18	0.67 (0.03)	0.18	0.65 (0.03)	0.18	0.66 (0.03)	0.18	0.75 (0.03)	0.18	0.79 (0.03)
**ssGBLUP + wG + QTN:3-fold**	0.20	0.62 (0.03)	0.19	0.60 (0.03)	0.19	0.61 (0.03)	0.20	0.71 (0.03)	0.20	0.77 (0.03)
**ssGBLUPrecords + wG + QTN:1-fold**	0.31	1.02 (0.02)	0.31	1.00 (0.02)	0.31	1.02 (0.02)	0.29	1.11 (0.2)	0.30	1.08 (0.02)

Prediction ability (Acc) and regression coefficient (b) for weighted single-step GBLUP (ssGBLUP+wG) including selected variants (PFV) in the model and applying different weighting approaches for PFV (1-fold, 2-fold and 3-fold the maximum weighted obtained in the ssGWAS)

## Discussion

Determination of the PFVs of complex phenotypes in livestock species is scarce and imperative. Some research groups have been trying to find the best way to identify PFVs, but they weren’t able to state whether these variants were causal or not and what were their full biological consequences for the phenotype [25–28]. Herein, we identified PFVs considering their biological effects and validated these findings by demonstrating the increased prediction accuracy for genomic selection by using such information in an independent population. Our approach used a systems biology rationale coupled with multi-tissue deep phenotyping and the effects on protein consequences of the PFVs for a given phenotype, in this case the feed efficiency in beef cattle.

The strategy used in this study consists of two major stages. The first is the identification of the PFVs using a systems biology approach, and the second is their validation through genomic selection. In the first stage, the GATK was used to call the variants: it compares the case RNA sequencing data with the control (bovine reference genome) using powerful filtering and statistical tools. This tool is widely used in several research to identify variants [29–32], and it is commonly used with the HaplotypeCaller algorithm, which improves performance by making the tool more accurate [33]. To select a variant calling tool, one needs to have a good combination of processing time, precision, and sensitivity (call quality) of the genotyping. When comparing GATK with other tools (i.e., Findvar, SAMtools, and Graphtyper) that display similar functions and considering the processing time, the GATK is at a disadvantage [33, 34]. However, when comparing the number of polymorphic sites found by the tools (homozygous and heterozygous), the GATK is of great advantage [34, 35]. Regarding the call for false positives, the tool with the lowest percentages was Findvar, followed by GATK, and SAMtools [34]. Although the GATK has a long processing time, it can be compensated by the number of polymorphic sites and the median filtering of false positives, making it a balanced tool for identifying the PFVs.

The second stage, on the other hand, involves validating the results obtained in the first stage using the genomic prediction in an independent population by including differential weighting in genomic regions harboring such PFVs on the prediction accuracy and inflation for RFI. The GWAS is generally used to find DNA variants associated with phenotypic variation of complex traits, however, it does not identify whether the variant is causal or not. Studies [36–39] point out that the use of *expression quantitative trait loci* (eQTL) mapping can help identify causal variants and also in the distinction between pleiotropic and binding effects [38]. It should be highlighted that a denser SNP panel is needed to accurately locate mutations and the genes involved, making eQTL studies very expensive. A viable alternative is a methodology that we performed herein: a systems biology-based characterization of the phenotype to detect the PFVs and further use as additional information for genomic prediction. Most importantly, several livestock phenotypes already have RNA-seq data publicly available. In this study, we used data from the PFVs from an RNA-seq analysis, which can serve as a tool to improve genetic predictions. Thus, the two factors (GWAS + PFVs) differentially weighted and added together increase the ability of genomic prediction. Adding external information from the PFVs identified through analysis of RNA-seq as well as those from the WssGWAS contributes to reducing selection risk by improving the GEBV accuracies, however, more inflated predictions were obtained as the weight for genomic information and PFV increased. The prediction ability for RFI was close to those obtained in previous studies using taurine [40, 41] and indicine [23, 24] cattle breeds. By comparing the prediction inflation from different tissues, it was possible to observe that the liver and muscle displayed less inflated predictions when compared to the adrenal glands, pituitary, and hypothalamus. These results indicate that the PFVs present in the liver and muscle contribute to more informative DNA variants than the other tissues. Although RNA-seq is not recommended for genotyping, this systems-biology approach using RNA-seq data detected DNA functional variants that improved genomic selection, showing the benefits to select the most predictive variants in coding regions, where MAF is often low and LD between mutations within a gene is high.

Gains in prediction accuracy are expected when widely known candidate regions identified by the GWAS are included and weighted in the prediction models [42]: (i) a total of 1623 variants from different cattle breeds where added to a custom SNP chip, and an accuracy gain of 2% was found when more weight was assigned to the QTN (Quantitative Trait Nucleotide) [43]; (ii) the addition of selected sequence variants from a multiracial GWAS generated an increase of up to 10% in accuracy [44] and; (iii) the incorporation of potential causative SNPs and removal of adjacent SNPs increased the accuracy by 2.5% in … [45]. Thus, the results obtained in our validation study pointed out that incorporating data derived from the PFVs improved the genomic prediction for RFI and increases the probability of pick-up a genetic marker in strong linkage disequilibrium with causal mutations. Further, it provides higher contribution of these SNP markers to the additive genetic variance for RFI.

Our analysis also allowed us to perform a functional enrichment analysis encompassing the genes with the PFVs. Over-represented terms for Class I MHC mediated antigen processing and presentation were described for all organs, along with the DNA double-strand break response specifically in the hypothalamus. An overrepresentation of the BOLA family genes and its polymorphisms (BOLA-DQA5, BLA-DQB, BOLA-DQA1, BoLA (ENSBTAG00000005182), JPS.1 (also known as BoLa), and BOLA-NC1) was found. These genes are mainly involved in the MHCclass I (JSP.1, BOLA, BoLA (ENSBTAG00000005182), and BOLA-NC1) and class II (BLA-DQB, and BOLA-DQA5). The MHC is a fundamental mechanism of the immune system, which has several functions and the response against infectious diseases is the most important [46, 47]. The MHC is divided into three groups: class I, class II, and class III. Class I molecules have as their main function to introduce peptides to CD8 + T lymphocytes, which in turn kill cells infected with viruses and neoplasms [48]. Class II molecules have direct and indirect functions, which include participation in antigen-presenting cells (APCs), such as dendritic and macrophage cells. These APCs have antigens derived from extracellular CD4 + T cell pathogens, which in turn activate macrophages and B cells to generate inflammatory and antibody responses, respectively. Indirectly, class II molecules participate in the immune process through steroid 21-hydroxylase enzymes and tumor necrosis factors [48]. In the MHC class I there are two subclasses: classic MHC-I (MHC-Ia) and non-classic (MHC-Ib). The BOLA-NC1 gene participates in the non-classical pathway, responsible for generating membrane isoforms from alternative splicing (differential splicing) [49–51]. In humans, the BOLA-NC1 gene is responsible for secreting molecules that interact with inhibitory receptors expressed by natural killer cells (NK), T lymphocytes, and APC to inhibit cells [52–60]. The MHC Class II can also be classified in two groups: DR and DQ [61]. BLA-DQB and BOLA-DQA5 participate in the DQ group, which is highly polymorphic and mainly acts in decreasing the response in CD4 helper T cells [62, 63]. These genes have already been reported in other studies such as bovine leukemia virus [61], comparison of MHC class II diversity between different cattle breeds [64], how the bovine MHC influences disease function and susceptibility [65], and bacterial infection and inflammation in dairy cattle [66, 67].

An increasing number of studies have demonstrated the link between the immune system and FE in different livestock species. In one of our previous studies [13], the transcriptomic analysis indicated that LFE animals had more periportal liver lesions and pronounced inflammatory response, which is mediated by the immune system. We also demonstrated that LFE animals have increased bacterial load, which is at least, in part, responsible for the hepatic lesions and inflammation in such animals [17]. Therefore, the identification of PFVs for FE in beef cattle in genes related to immune response is very plausible and opens the possibility for fast improvement through genetic selection of this important phenotype in cattle.

## Conclusion

In conclusion, the identification of PFVs by a systems biology based on multi-organ deep phenotyping by RNA-seq data increased the accuracy of the prediction for young animals without phenotypic records. The study also identified that variants existent in the liver and muscle have more impact on genomic predictions for young animals, highlighting the importance of these two organs for feed efficiency. The PFVs identified herein were found to be mainly involved in the processing and presentation of MHC class I and II mediated antigens, an important mechanism of the innate immune system. It did not escape our attention that this strategy for detecting PFVs for genetic selection can be used for other livestock species since the main biological pathways of feed efficiency are similar for other species, such as pigs, poultry, and dairy cows.

## Methods

### Phenotypic data and biological sample collection

All animal protocols were approved by the Institutional Animal Care and Use Committee of Faculty of Food Engineering and Animal Sciences, University of São Paulo (FZEA-USP – protocol number 14.1.636.74.1). All procedures to collect the phenotypes and biological samples were carried out at FZEA-USP (Pirassununga, State of São Paulo, Brazil). Ninety-eight Nellore bulls (*Bos taurus indicus*) (16 up to 20 months of age and 376 ± 29 kg BW) were evaluated in a feeding trial which comprised 21 days of adaptation to the feedlot diet followed by a 70-day period of data collection. Total mixed feed was offered ad libitum and daily dry matter intake (DMI) was individually measured. Animals were weighted at the beginning, at the end, and every 2 weeks during the experiment period. Feed efficiency was estimated by residual feed intake (RFI) [68]. Forty animals selected either as high feed efficiency (HFE) or low feed efficiency (LFE) groups were slaughtered on 2 days with a 6-day interval. Samples from liver, muscle, hypothalamus, and pituitary and adrenal glands were collected from each animal at the slaughter and were quickly frozen in liquid nitrogen and stored at − 80 °C. Further information about the management and phenotypic measures of the animals used in this study can be found elsewhere [13].

### RNA-seq data

Samples of nine animals from each FE group (high and low) were selected for RNA-seq using RFI [13]. The total RNA from liver, muscle, adrenal, pituitary, and hypothalamus samples was extracted by using the RNeasy mini kit (QIAGEN, Crawley, West Sussex, UK) according to the instructions provided by the manufacturer. The total RNA quality and quantity were assessed using automated capillary gel electrophoresis on a Bioanalyzer 2100 with RNA 6000 Nano Labchips according to the manufacturer’s instructions (Agilent Technologies Ireland, Dublin, Ireland). Samples that presented RNA integrity number (RIN) less than 8.0 were discarded. The mRNA libraries were constructed using the TruSeq™ Stranded mRNA LT Sample Prep Protocol and sequenced on Illumina HiSeq 2500 equipment in a HiSeq Flow Cell v4 using HiSeq SBS Kit v4 (2x100pb). FastQC software (Babraham Institute, Cambridge, UK, http://www.bioinformatics.babraham.ac.uk/projects/fastqc/) was used to visualize the sequencing quality. The removal of Poly A/T tails and adapters was carried out using the Seqyclean software (University of Idaho: Institute of Bioinformatics and Evolutionary Studies, Moscow, USA, https://bitbucket.org/izhbannikov/seqyclean), and bases with quality ≥20 and complete reads with at least 50 bp were considered for subsequent analysis. Alignment of the reads was done using the STAR software version 2.7 [69] with the reference genome *Bos taurus* ARS-UCD1.2 (Ensembl, ftp://ftp.ensembl.org/pub/release-98/fasta/bos_taurus/dna/), allowing two mismatches per read.

### Protocol to call the potential functional variants associated with FE

Genome Analysis Toolkit (GATK) software version 4.0.11.0 [70] was used to call the variants. The HaplotypeCaller command was applied to identify the variants (Single Nucleotide Polymorphism - SNPs) and insertions and deletions (INDELs). The variants underwent quality control on the GATK software as follows: (Variant Quality Score - QUAL) > 30, depth of sequencing (Deph Plot - DP) > 4, amount of available coverage (QualByDepth - QD) > 3, polarization trend (FisherStrand - FS) > 30, and the general mapping quality of readings that support a variant call (RMSMappingQuality- MQ) < 35. Statistical analysis was carried out using Plink software [71] considering MAF “SNP with” < 40% and a call rate “>” 50%. For the allele frequency test between HFE and LFE groups “without assuming the Hardy-Weinberg equilibrium”, the Cochran-Armitage “allelic test” was used considering significant differences when *P* < 0.05.

### Characterization of the effects of variants on protein sequence and function

The PFVs were analyzed in the Variant Effect Predictor (VEP) online tool release 98 [72], which predicts the functional effects of the variants. VEP indicates the type of impact, which can be “high”, causing protein truncation and loss of function; “moderate” as a non-disruptive variant that can alter the protein’s effectiveness; or “low”, unlikely to alter the behavior of proteins; “type modifier”, non-coding variants or variants that affect non-coding genes, in which predictions are difficult or there is no evidence of impact. In this strategy, only high and moderate impacts were considered.

### Functional enrichment analysis

The functional enrichment analysis was carried out using DAVID version 6.8 [73] to identify over-represented biological pathways in the set of genes with PFVs. The type of analysis was the “overrepresentation test” using the list of genes (*n* = 5857) with PFVs identified from the RNA-seq of the five selected tissues (liver, muscle, pituitary gland, hypothalamus, and adrenal gland). For the statistical analysis, the bonferroni, benjamini and False Discovery Rate (FDR) test was carried out. Significant pathways were considered when FDR < 0.05.

### Validation of the potential functional variants by genomic prediction

#### General information about the data

The validation of the PFVs for the RFI obtained in different five tissues was performed in an independent Nellore cattle population. The influence of differential weighting in genomic regions harboring the PFVs on the prediction accuracy and inflation for the RFI was evaluated. The validation of the PFVs was performed by including differential weighting in genomic regions associated with RFI identified by the weighted single-step GWAS (WssGWAS) analysis, together with candidate regions harboring PFVs obtained in this study. To calculate the genetic merit and the SNP effects, the SNPs weights were estimated by performing a WssGWAS. In this methodology, the SNP weights are obtained iteratively. The iterative process increases the weights of the SNP with large effects and decrease those with small effects, essentially regressing them to the mean [74]. Such methodology leads to gains in QTLs detection by revealing genomic regions that accounted for a higher portion of the additive genetic variance [74, 75].

Records for RFI were obtained from the Nellore Brazil breeding program coordinated by the National Association of Breeders and Researchers (ANCP, Ribeirão Preto-SP, Brazil) from feed efficiency tests carried out between 2011 and 2018. A total of 4653 phenotyped and 5117 genotyped animals collected in 60 feed efficiency tests in the feedlot system using the same protocol as described by [22] were used. There were 2065 animals with phenotype and genotypic information in the dataset.

The animals were evaluated under similar management and environmental conditions in the feedlot with an average of 423 ± 122 days of age at the beginning of the tests. During the tests, the animals were exposed to a feeding trial which comprised 21 days of adaptation to the feedlot diet followed by 70 days of data collection. The average weight of each animal was obtained by periodic manual weighing or by automated weighing platforms (GrowSafe® or Intergado®).

The following feed intake records were not considered in the analyses: days when the animals were handled outside the facilities for several hours, equipment failure, and when no refusals were found. The dry matter percentage was determined from weekly samples of the offered diet and refusals. The average daily gain (ADG) in each test was considered as the linear regression coefficient of the body weight on days in the test (DIT):

$${\textrm{y}}_{\textrm{ij}}=\upalpha +\upbeta \ast \textrm{DIT}+{\upvarepsilon}_{\textrm{i}}$$

where, *y*_*i*_ is the weight of the i^*th*^ animal on the j^th^ day; *α* is the intercept of the regression equation which represents the initial weight; *β* is the linear regression coefficient which represents the ADG; DIT_i_ is the day in the performance test of the i^*th*^ observation; and ε is the error associated with each observation.

The DMI (kg/day) was obtained by calculating the average daily intake values during the test period. In individual stalls, this parameter was calculated as the difference between the dry matter offered and the refusal. In group pens, the DMI was calculated from the amount of individually consumed feed automatically recorded by the electronic systems.

Metabolic weight MW (kg^0.75^) was retrieved from the liveweight and ADG as follows:

$${MW}_i={\left[\alpha +\beta \ast \left(\frac{DIT}{2}\right)\right]}^{0.75}$$

where *MW*_*i*_ is the metabolic weight of the i^*th*^ animal; *α* is the intercept of the regression equation which represents the initial weight; DIT as described above; and *β* is the linear regression coefficient which represents the ADG, as described and obtained above in estimating ADG. RFI (kg of DM/day) was estimated within each contemporary group (CG) by the residual of the DMI regression as a function of ADG and MW, using a multiple regression model regressing DMI on ADG and MW, in the following model:

$${y}_i={\beta}_o+{\beta}_1 ADG+{\beta}_2 MW+{\beta}_3 CG+{\varepsilon}_i\ (RFI)$$

where *y*_*i*_ is the individual DMI of the i^*th*^ animal; β1, β2, and β3 are the linear regression coefficient of ADG, MW and CG, respectively; and *ε*_*i*_ is the residual error of the i^*th*^ animal (i.e., RFI).

The relationship matrix used in the WssGWAS and prediction analyses was calculated based on pedigree information from 19,507 animals with 1809 sires and 9147 dams through nine generations, provided by the Nellore Brazil Breeding Program, coordinated by the ANCP. More information regarding the set of animals used in this study can be found elsewhere [22].

#### Genomic information

A total of 963 animals were genotyped using the Illumina BovineHD BeadChip (Illumina Inc., San Diego, CA, USA), which contains 777,962 SNP markers of an independent population. These animals were used as a reference population to impute genotypes of 5117 animals, previously genotyped with a low-density panel (CLARIFIDE® Nelore 3.1) encompassing over 27,000 SNP markers. Genotype imputation was carried out using the FImpute 2.2 software [76]. Quality control criteria were carried out using the PREGSF90 package [77], removing animals and markers with a call rate < 0.90 and minor allele frequency (MAF) < 0.05. Monomorphic SNPs with redundant position and those located on non-autosome chromosomes were removed. Additionally, animals and SNPs with Mendelian conflicts were excluded.

#### Weighted single step genome-wide association study

To carry out the GWAS, the dataset was split into training (*n = 3253* animals) and validation (*n = 1864* animals) subsets. The validation dataset was composed of young animals without progeny records with genotypic and phenotypic information. The training dataset was composed of genotyped and phenotypes sires with phenotyped progenies. In the training population, a total of 201 genotyped and phenotyped animals and 2588 ungenotyped and phenotyped animals were described. The phenotypes and genotypes of the validation subset were omitted in the GWAS. The GWAS analysis was carried out using the training subset and applying the weighted single-step GWAS (WssGWAS) methodology [74]. The WssGWAS was carried out to estimate the weights for SNPs markers iteratively (*n = 2*).

The animal linear model included the fixed effects of contemporary group (CG) and the animal age as covariable, and the random direct additive genetic effect. The CG were composed of farm, management group, sex, feed efficiency test, year, and birth season. Records within ±3.5 standard deviations from the CG mean were considered in the analysis, and the CG that had at least four animals were also considered in the analysis. The animal model used was:

$$\textbf{y}=\textbf{X}\boldsymbol{\upbeta } +\textbf{Zu}+\textbf{e}$$

where **y** is a vector of phenotypic records; **β** is a vector of fixed effects, including the CG and age at calving; **X** is the incidence matrix associating **β** with **y**; **u** is a vector of random effects of the direct additive genetic effects; **Z** is the incidence matrix associating with **y**; **e** is the residual effect. Assumptions for residual effects are described below:

$$\textbf{e}\sim \textbf{N}\left(\textbf{0},\textbf{I}{\boldsymbol{\upsigma}}_{\textbf{e}}^{\textbf{2}}\right),$$

where ${\boldsymbol{\sigma}}_{\boldsymbol{e}}^{\textbf{2}}$ is the residual variance, and **I** is an identity matrix with a dimension equal to the number of animals. The ssGBLUP method was used with $\textbf{a}\sim \textrm{N}\left(\textbf{0},\textbf{H}{\upsigma}_{\textrm{a}}^2\right)$, where **H** is defined as in Legarra et al. (2009) and its inverse is the same as in BLUP [78]:

$${\textbf{H}}^{-\textbf{1}}={\textbf{A}}^{-\textbf{1}}+\left[\begin{array}{cc}\textbf{0}& \textbf{0}\\ {}\textbf{0}& {\textbf{G}}^{-\textbf{1}}-{\textbf{A}}_{\textbf{22}}^{-\textbf{1}}\end{array}\right]$$

where ${\textbf{A}}_{\textbf{22}}^{-\textbf{1}}$ is the inverse of the numerator relationship matrix for the genotyped animals, and **𝐆** is genomic relationship matrix.

The **G** matrix was obtained following [79]:

$$\textbf{G}=\frac{\left(\textbf{M}-\textbf{P}\right){\left(\textbf{M}-\textbf{P}\right)}^{\prime }}{2{\sum}_{\textrm{j}=1}^{\textrm{m}}{\textrm{p}}_{\textrm{j}}\left(1-{\textrm{p}}_{\textrm{j}}\right)}$$

where **M** is an allele-sharing matrix with *m* columns (*m* total number of markers) and *n* rows (*n* = total number of genotyped individuals), and **P** is a matrix containing the frequency of the second allele (*pj*), expressed as 2*pj*. M*ij* was 0 if the genotype of individual *i* for SNP *j* was homozygous AA, was 1 if heterozygous, or 2 if the genotype was homozygous BB. To account for heterogeneous SNP weights, a matrix of weights should be included in the formula for constructing **G**, in which *var(s)* is the vector containing the variance of the individual SNP effects, and *d*_*i*_ is the _*i*_*th* diagonal element of **D**, accounting for the _*i*_*th* SNP weight:

$$\mathit{\operatorname{var}}(s)=\textbf{D}=\left|\begin{array}{ccc}{d}_1& 0& 0\\ {}0& {d}_2& 0\\ {}0& 0& {d}_n\end{array}\right|$$

Based on that, a weighted relationship matrix can be defined as:

$${\textbf{G}}_{\textbf{w}}=\frac{\operatorname{var}\ \left({\textrm{a}}_{\textrm{g}}\right)}{\upsigma_{\textrm{a}}^2}=\frac{\operatorname{var}\left(\textrm{Zu}\right)}{\upsigma_{\textrm{a}}^2}={\textbf{ZDZ}}^{\prime}\boldsymbol{\uplambda}$$

The **𝜆** is a ratio of variances or normalization constant (Vanraden et al., 2009):

$$\boldsymbol{\lambda} =\frac{\sigma_u^2}{\sigma_a^2}=\frac{1}{\sum_{i=1}^m2{p}_i\left(1-{p}_i\right)}$$

where 𝒎 is the number of SNPs and 𝒑_𝒊_ is the allele frequency of the second allele of the *i-th* SNP. According to Stranden & Garrick [80], the SNPs effect (**û**) can be obtained as follows:

Estimates of the SNP effects can be used to estimate the individual variance of each SNP effect (${\upsigma}_{\textrm{u},\textrm{i}}^2$), and apply a different weight to each SNP as follows:

$${\upsigma}_{\textrm{u},\textrm{i}}^2={\textrm{u}}_{\textrm{i}}^22{p}_i\left(1-{p}_i\right)$$

In summary, the SNP effects and weights obtained in the WssGWAS were derived as follows [81]:

1. Let D = I in the first step.
2. Calculate G = **ZDZ**^′^**λ.**
3. Calculate GEBVs for the entire dataset using the ssGBLUP.
4. Convert GEBVs to SNP effects $\left(\hat{u}\right):\hat{u}=\boldsymbol{\lambda}\ {DZ}^{\hbox{'}}{\left({ZDZ}^{\hbox{'}}\boldsymbol{\lambda} \right)}^{-1}{\hat{\textrm{a}}}_{\textrm{g}}$, where ${\hat{\textbf{a}}}_{\textbf{g}}$ is the GEBVs of animals which were also genotyped.
5. Calculate the weight for each $\textrm{SNP}:{d}_i={{\hat{u}}^2}_i2{p}_i\left(1-{p}_i\right)$, where *i* is the *i-th* SNP.
6. Normalize the SNP weights to keep the total genetic variance constant.

The SNP weights were calculated iteratively (*n = 2*) looping through steps 2–6.

#### Prediction models

To evaluate the impact of causative PFVs on RFI genomic predictions, the same model applied for the WssGWAS analyses was applied. The genomic breeding value (GEBV) of the validation subset was calculated considering the whole population (training + validation subsets) by applying the weighted single-step genomic BLUP procedure (WssGBLUP). The WssGBLUP is a weighted adaptation proposed to predict the genomic values [74], which is based on an iterative process with weights to update the SNP solutions.

To evaluate the impact of the causative PFVs on RFI genomic predictions, the G matrix was constructed using different combinations of SNPs and weights: (a) Unweighted G matrix with 460,992 SNPs; (b) weights in D calculated based on genome-wide association studies (ssGWAS) using iterative WssGBLUP as in [75], updating the GEBV and SNPs weights for 2 iterations; c) weighted SNPs as b) and also including differential weights for SNPs neighboring the causative PFVs for liver, adrenal, pituitary, hypothalamus and muscle tissue, respectively. The inclusion of causative PFVs for five tissues were carried out by weighting the SNPs adjacent to causative PFVs since the SNPs in linkage disequilibrium with causal variants received higher weights. In this sense, the maximum weights (lambda values) estimated in the ssGWAS after two iterations were used to weight the SNPs adjacent to the causative PFVs. Weighting the SNPs adjacent to the regions with the highest value of diagonal element of **D** matrix updates the model, in which these regions exhibit higher influence for the trait compared to other genomic regions. Hence, three levels of weight for SNPs neighboring the PFVs were tested, 1-fold, 2-fold, and 3-fold the maximum weighted (**D** matrix diagonal element) obtained in the ssGWAS after two iterations. The prediction analyses were performed using the BLUPF90 software family [82] including the genomic information [81].

The prediction accuracies were calculated according to the Beef Improvement Federation (BIF) [83] as follows:

$${\textrm{Acc}}_{\textrm{BIF}}=1-\sqrt{\frac{\textrm{PEV}}{\left(1+{\textrm{F}}_{\textrm{i}}\right)\times {\upsigma}_a^2}}$$

where PEV is the prediction error variance, ${\upsigma}_a^2$ the additive genetic variance, and F_i_ the inbreeding coefficient. The regressions coefficient between the GEBV obtained using the complete dataset considering the unweighted **G** and the GEBV estimated for different weighted G scenarios with or without PFVs was used to evaluate the prediction inflation.

## Supplementary Information

**Additional file 1: Table S1.** Mean and standard deviation (SD) of tissue alignment results.

**Additional file 2: Table.** Biological pathways enriched from potential functional variants associated with feed efficiency by tissue.

## Data Availability

The dataset supporting the results of this article is available in the European Nucleotide Archive (ENA) as part of FAANG consortium under the study ID PRJEB27337. [https://www.ebi.ac.uk/ena/browser/view/PRJEB27337?show=reads].

## Abbreviations

ANCP: *Associação Nacional de Criadores e Pesquisadores*
APCs: Antigen-presenting cells
BIF: Beef Improvement Federation
BLUP: Best Linear Unbiased Prediction
CG: Contemporary Group
DMI: Dry Matter Intake
DP: Depth Plot
EBV: Estimated breeding values
eQTL: Expression Quantitative Trait Loci
FDR: False Discovery Rate
FE: Feed Efficiency
FindVar: Find Variables by Name
FS: Fisher Strand
GATK: Genome Analysis Toolkit
GBLUP: Genomic Best Linear Unbiased Prediction
GEBV: Vector of Genomic EBV
GWAS: Genome-Wide Association Studies
HFE: High Feed Efficiency
INDELs: Insertion and Deletions
LFE: Low Feed Efficiency
MAF: Minor Allele Frequency
MHC: Major Histocompatibility Complex
MQ: RMSMapping Quality
PEV: Position-effect variegation
PFVs: Potential Functional Variants
QD: Quality by Depth
QTL: Quantitative Trait Locus
QTN: Quantitative Trait Nucleotide
QUAL: Variant Quality Score
rec: RFI records
RFI: Residual Feed Intake
RIN: RNA Integrity Number
RNA-seq: RNA-sequencing
SAMtools: Sequence Alignment/Map
SIFT: Scale-Invariant Feature Transform
SNP: Single Nucleotide Polymorphisms
ssBLUP: Single step Best Linear Unbiased Prediction
ssGBLUP: Single step Genomic Best Linear Unbiased Prediction
ssGWAS: Single-step GWAS
STAR: Spliced Transcripts Alignment to a Reference
VEP: Variant Effect Predictor
wG: Weighted G matrix
