# Supplementary material for: Detection of potential functional variants based on systems-biology: the case of feed efficiency in beef cattle
Source: BMC Genomics. 2022 Nov 25;23:774. doi: 10.1186/s12864-022-08958-y (PMC9700932; doi:10.1186/s12864-022-08958-y)
Supplement: Supplementary file 1 — Additional file 1: Table S1. Mean and standard deviation (SD) of tissue alignment results. [file 12864_2022_8958_MOESM1_ESM.pdf]

Table S1. Mean and standard deviation (SD) of tissue alignment results

Tissue	Number of initial reads*	% Reads mapped only	% Reads mapped to multiple loci	% Reads unmapped	% Reads unmapped: other	% Coverage
**Adrenal**	14,133,167 ± 1,457,849	82 ± 4	4 ± 0,3	14 ± 4	0,03 ± 0,01	86 ± 4
**Hypothalamus**	13,309,273 ± 1,905,237	81 ± 5	2 ± 0,1	16 ± 5	0,07 ± 0,02	84 ± 5
**Muscle**	13,205,295 ± 1,045,274	87 ± 3	2 ± 0,2	11 ± 3	0,08 ± 0,01	89 ± 3
**Liver**	13,332,857 ± 1,149,619	69 ± 16	6 ± 2	25 ± 15	0,04 ± 0,02	75 ± 15
**Pituitary**	12,512,647 ± 1,440,560	91 ± 1	3 ± 0,1	6 ± 1	0,06 ± 0,01	94 ± 1

* these values are not in percentage; they are in number of reads
